# Hydrogen peroxide-independent generation of superoxide by plant peroxidase: hypotheses and supportive data employing ferrous ion as a model stimulus

**DOI:** 10.3389/fpls.2014.00285

**Published:** 2014-07-01

**Authors:** Makoto Kimura, Yosuke Umemoto, Tomonori Kawano

**Affiliations:** Laboratory of Chemical Biology and Bioengineering, Graduate School and Faculty of Environmental Engineering, The University of KitakyushuKitakyushu, Japan

**Keywords:** alkalization, auxin, Compound III, oxidative burst, peroxidase, superoxide

## Abstract

When plants are threaten by microbial attacks or treated with elicitors, alkalization of extracellular space is often induced and thus pH-dependent extracellular peroxidase-mediated oxidative burst reportedly takes place, especially at the site of microbial challenge. However, direct stimulus involved in activation of peroxidase-catalyzed oxidative burst has not been identified to date. Here, we would like to propose a likely role for free ferrous ion in reduction of ferric native peroxidase into ferrous enzyme intermediate which readily produces superoxide anion *via* mechanism involving Compound III, especially under alkaline condition, thus, possibly contributing to the plant defense mechanism. Through spectroscopic and chemiluminescence (CL) analyses of reactions catalyzed by horseradish peroxidase (HRP), the present study proposed that plant peroxidase-catalyzed production of superoxide anion can be stimulated in the absence of conventional peroxidase substrates but in the presence of free ferrous ion.

## Introduction

In plants, two major mechanisms leading to the production of reactive oxygen species (ROS) involving either NADPH oxidases or peroxidases (POXs) have been proposed (Yoshioka et al., 2008). Events of plant defense against pathogenic microorganisms, represented by plant cellular perception of microbial molecules contributing to so-called microbe-associated molecular patterns (MAMPs) such as bacterial flagellin, referred to as pattern-triggered immunity, reportedly trigger a rapid and transient accumulation of ROS (O'Brien et al., 2012a). In *Arabidopsis*, molecular evidence for involvement of two identified cell wall POXs, namely, PRX33 and PRX34, in MAMPs-responsive apoplastic ROS generation has been reported (Bindschedler et al., 2006; O'Brien et al., 2012a,b).

In fact, plants are rich sources of enzymes involved in production and removal of ROS (Yoshioka et al., 2008). A Swiss group of POX research specialists metaphorically described that plant enzymes belonging to POXs (EC 1.11.1.7) display more functions than a “Swiss army knife” (Passardi et al., 2005). As suggested, highly diversified roles of plant POXs including regulation of hydrogen peroxide (H_2_O_2_) level, oxidation of various substrates, generation of ROS coupled to oxidation of aromatic monoamines (AMAs) such as phenylethylamine (Kawano et al., 2000a,b) and phenolics such as salicylic acid (SA) (Kawano et al., 2004; Kawano and Bouteau, 2013) in living plants have been documented to date. By using a variety of electron (e^−^)-donating substrates and H_2_O_2_, the common e^−^ acceptor, plant POXs achieve a great deal of oxidation reactions essential for the functions of living cells (Kawano, 2003a). Through production of certain POX isoforms at specific timing and localization, thus by properly and precisely making use of a variety of plant POX functions, the growing plants can respond to and combat a wide variety of stressful challenges with biotic or abiotic nature (Penel, 2000; Hiraga et al., 2001).

**Figure 1:**
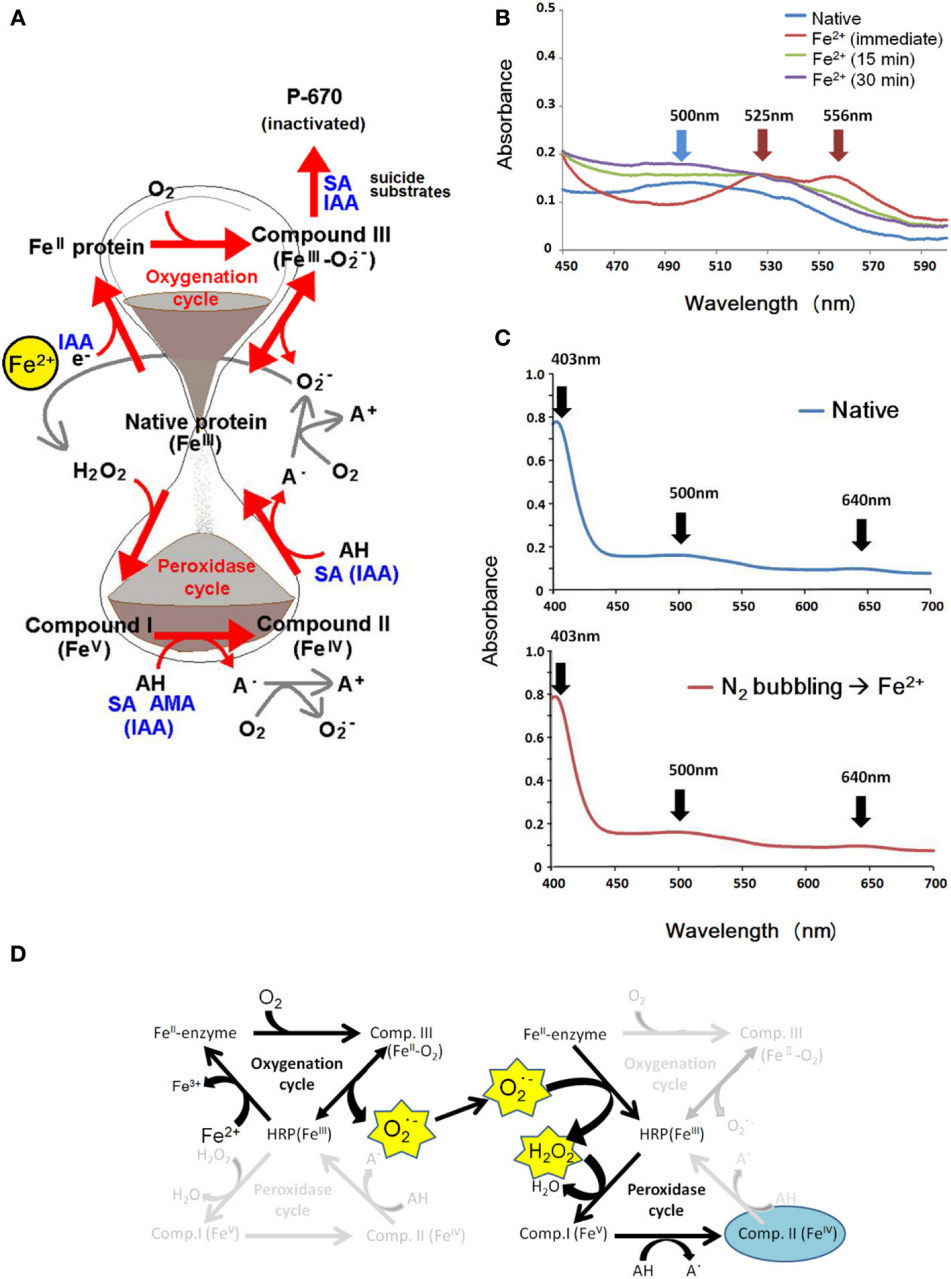
**Effects of key substrates for plant POXs on the inter-conversion of enzyme intermediates differed in redox-status. (A)** Summary of known reactions involving IAA, SA, and AMAs leading to spectroscopic changes reflecting the presence of native ferric enzyme (absorption peaks at 403, 500, and 639 nm), ferrous enzyme (absorption maxima at 438 and 580 nm), Compound I (absorption maxima at 577, 622, and 650 nm), Compound II (absorption maxima at 420, 527, and 556 nm) and Compound III (absorption maxima at 545 and 578 nm). In this article, ferrous ion is proposed as the stimulus converting native enzyme to the ferrous enzyme complex. **(B)** Effect of Fe^2+^ on dissolved O_2_-dependent conversion of native HRP into Compound II. To the reaction mixture (final total volume, 400 μl; prior to Fe supplementation, 383 μl) contained K-phosphate (25 mM, pH 7.0), HRP (15 μM), 17 μl of 5 mM Fe^2+^ (as FeSO_4_, final conc., 200 μM) was added. Then sample was spectroscopically scanned within 1 min (immediate) and at 15 and 30 min after addition of Fe. **(C)** Similarly to **(B)**, to the reaction mixture (final total volume, 1 ml; prior to Fe supplementation, 940 μl) containing K-phosphate (25 mM, pH 7.0) and HRP (15 μM) and presence of native enzyme was spectroscopically confirmed (top). To the above reaction mixture, Fe^2+^ was added (40 μl of 5 mM FeSO_4_; final conc., 200 μM), but only after deoxygenation with N_2_ gas (N_2_ bubbling) which was passed through using the syringe needle for 15 s (bottom). **(D)** The likely paths of Fe^2+^-induced conversion of native HRP to Compound II *via* of O^•^_2_ generating steps followed by release of H_2_O_2_. Note that the O^•^_2_-to-H_2_O_2_ conversion can proceed without enzyme at acidic condition and free Fe^2+^ may accelerate the processes.

As intensively discussed in the plant research community, oxidation of phenolics is one of the key functions of POXs (Passardi et al., 2005). It is widely accepted that, in the presence of H_2_O_2_, plant POXs can catalyze the generation of superoxide anion radical (O^•−^_2_) upon oxidation of substrates, chiefly phenolics (Kawano, 2003a; Yoshioka et al., 2008). Lower half of Figure 1A summarizes the mode of O^•−^_2_ production *via* e^−^ acceptor-dependently initiated conventional POX cycle enabling various substrates (AH) such as SA and AMAs. Apart from such H_2_O_2_-requiring reaction, plant POXs are also capable of O^•−^_2_ generation *via* e^−^ donor-dependently initiated and oxygen-requiring cycle involving few known substrates such as indole-3-acetic acid (IAA), the principal form of natural auxin in higher plants (Gazarian and Lagrimini, 1998; Savitsky et al., 1999; Kawano et al., 2001). Therefore, the O^•−^_2_-generating reactions catalyzed by plant POXs can be dissected into (i) the H_2_O_2_-dependent POX cycle and (ii) H_2_O_2_-independent oxygenation cycle as illustrated in Figure 1A. This model dissecting two distinct cycles initiated by interaction of native POX with e^−^ acceptor or e^−^ donor, is sometimes referred to as the hourglass model due to its shape (Kawano, 2003a; Takayama et al., 2012; Kawano and Bouteau, 2013).

We view here that the role of IAA in POX-catalyzed generation of O^•−^_2_ is one of effective e^−^ donors converting native enzyme into ferrous intermediate in the oxygenation cycle (Figure 1A, upper half). Idea on the IAA-dependent reduction of native plant POX to ferrous enzyme intermediate has been proposed by Smith et al. (1982). The series of reactions triggered by IAA further proceeds under the atmospheric condition rich in O_2_, therefore, the ferrous complex might be short-lived and readily converted to O_2_-bound form of enzyme intermediate known as Compound III (CIII) in which the state of heme iron can be described as O_2_-heme-Fe^II^ or O^•−^_2_-heme-Fe^III^ (Kawano et al., 2002a). Then, gradual decay of this complex into native enzyme at heme-Fe^III^ state accompanies the release of O^•−^_2_ (Figure 1A) as confirmed with IAA-stimulated horseradish peroxidase (HRP) using O^•−^_2_-specific chemiluminescence (CL) probe, *Cypridina* luciferin analog (CLA) (Kawano et al., 2001).

Assuming that the hypothetical model mechanism proposed in Figure 1A is correct, we should be able to screen or identify some effective e^−^ donors from a variety of single e^−^ reducing agents which target the native enzyme to trigger the onset of oxygenation cycle in plant POXs, eventually leading to a robust and long-lasting burst of O^•−^_2_ production. After testing a wide range of chemicals, we observed that free ferrous ion (Fe^2+^) acts as a novel inducer of O^•−^_2_ production in aid of plant POX, possibly by behaving as an effective e^−^ donor for Fe^III^-to-Fe^II^ conversion of heme in a model POX, HRP. The aim of the present article is to share our novel finding on the Fe-driven O^•−^_2_ production mechanism involving HRP.

## Materials and methods

### Chemicals

Purified HRP was purchased from Sigma (St. Louis, MO., USA), and used without further purification. CLA (2-methyl-6-phenyl-3,7-dihydroimidazo[1,2-a]pyrazin-3-one), a chemiluminescent probe for O^•−^_2_, was purchased from Tokyo Kasei Kogyo Co. (Tokyo, Japan). Luminol, SA, IAA, metals, and other chemicals except for enzyme were purchased from Wako Pure Chemical Co. (Osaka, Japan). IAA (100 mM) was first dissolved in ethanol and diluted to the desired concentrations with heated water (80°C). Then IAA solution was kept on ice in darkness until used. Final ethanol concentration in the reaction mixture was adjusted to be 0.1% (v/v).

### Spectroscopy

Concentration of HRP was determined spectroscopically by measuring the concentration of heme (ε 403 nm = 102 mM^−1^·cm^−1^) (Gazaryan et al., 1996). Changes in absorption spectra of HRP in 20 mM K-phosphate buffer (pH 7.0) were recorded on spectrophotometer (Shimadzu UV-1800, Kyoto, Japan) at room temperature with a spectral bandwidth of 1.0 nm in a cuvette with 1-cm light path. Compounds II (CII) and CIII derived from native HRP (7.5 μM) were determined spectroscopically.

### Chemiluminescence (CL) analysis

Generation of H_2_O_2_ and O^•−^_2_ in the HRP reaction mixture were monitored with H_2_O_2_-specific CL of luminol and O^•−^_2_-specific CL of CLA using a luminometer (Luminescensor PSN AB-2200-R, Atto Corp., Tokyo, Japan) and expressed as relative luminescence units (rlu) as previously described for HRP-catalyzed generation of ROS (Kawano et al., 2001).

## Results

### Preliminary spectroscopic analyses

Basically, formation of CII from native ferric POX in the conventional peroxidase cycle requires the presence of H_2_O_2_, as we have previously observed that addition of excess H_2_O_2_ to HRP reaction mixture readily results in transient formation of Compound I (CI) followed by increase in CII without supplementation of any additional molecules known as POX substrate such as phenolics or amines (Kawano et al., 2002c,d). However, we observed that addition of Fe^2+^ to HRP resulted in accumulation of CII without addition of exogenous H_2_O_2_ (Figure 1B), suggesting that H_2_O_2_ is formed after a series of reactions involving redox changes in POX/Fe^2+^ system. Interestingly, conversion of native HRP to CII by Fe^2+^ was completely inhibited by deoxygenating treatment such as bubbling with N_2_ gas (Figure 1C) and addition of sodium dithionite (data not shown), indicating the involvement of molecular oxygen at least at a certain step in the course of native-to-CI conversion.

### Iron-induced generation of ROS

**Figure 2:**
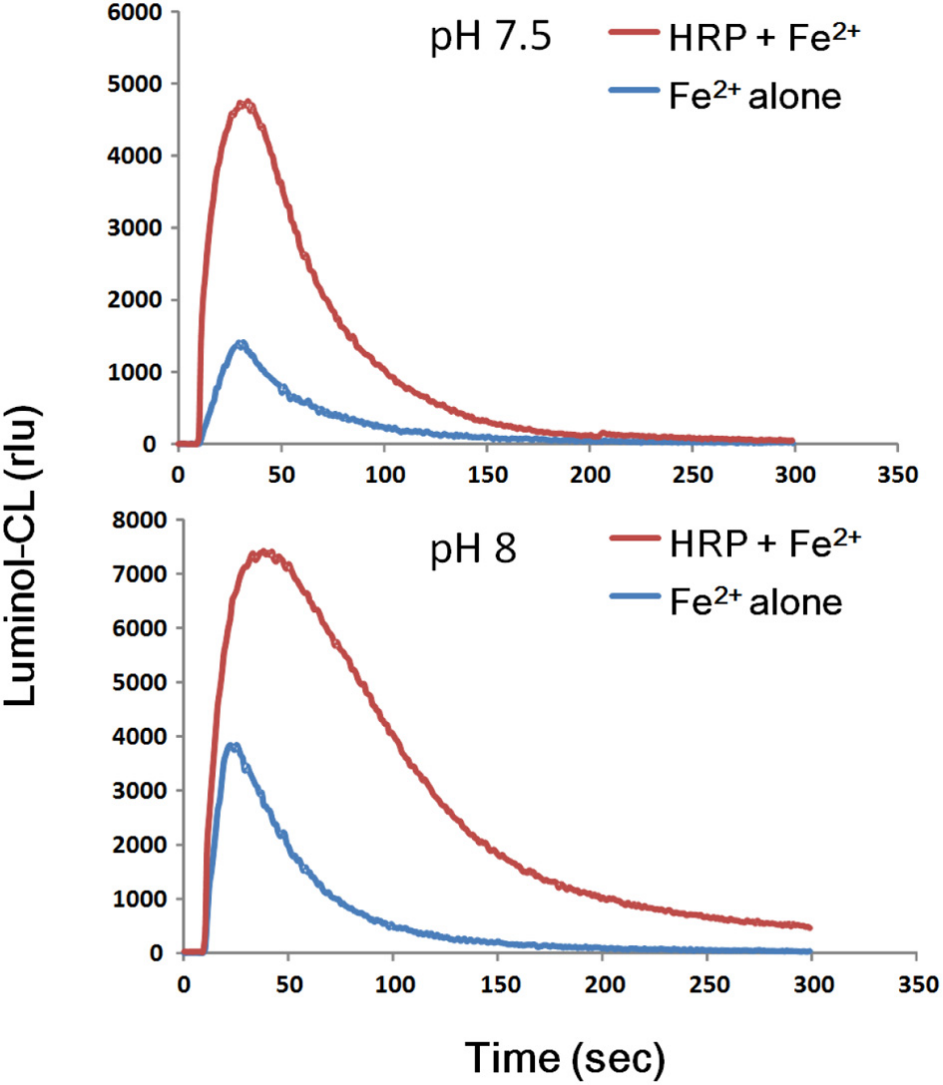
**Transient production of H_2_O_2_ following addition of Fe^2+^ to HRP reaction mixture**. Reaction mixture (0.2 ml) contained 25 mM K-phosphate (pH 7.5 or 8.0), 1.5 μM HRP, 10 μM luminol, and 50 μM FeSO_4_. For comparison, 50 μM FeSO_4_ was added to the mixture lacking HRP (Fe^2+^ alone). The resultant H_2_O_2_ might be used for further reactions converting native enzyme to unstable Compound I and eventually to Compound II.

As expected from the behavior of Fe^2+^ converting the native enzyme to CII, release of H_2_O_2_ could be detected in Fe^2+^-added HRP reaction mixture (Figure 2). Therefore, it is tempting to conclude that Fe^2+^-dependently produced H_2_O_2_ plays a key role in conversion of native enzyme to CII *via* transient formation of CI.

**Figure 3:**
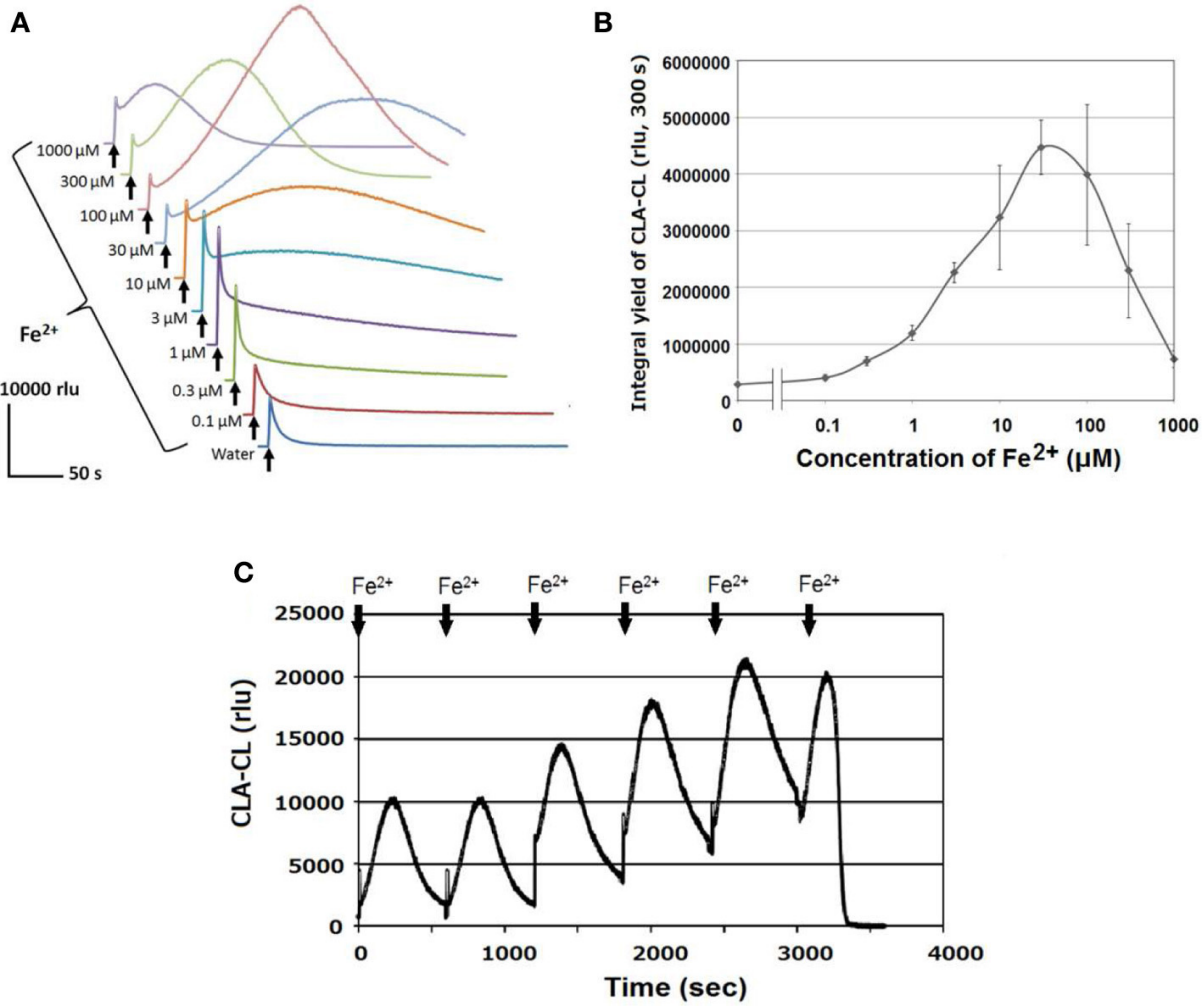
**Fe^2+^-induced O^•^_2_ generation in HRP reaction mixture. (A)** Temporal changes in Fe^2+^-induced O^•^_2_-dependent CLA-CL. **(B)** Effect of Fe^2+^ concentrations (as indicated) on the yield of CLA-CL summed up within 300 s after addition of Fe^2+^ or control water. Bars, SD (*n* = 3). **(C)** Successive addition of 50 μM Fe^2+^ until CLA run out. Conditions: total volume, 0.2 ml; K-phosphate, 25 mM (pH 7.0); HRP, 1.5 μM; CLA, 10 μM **(A,B)**, 40 μM **(C)**.

Similarly to the profile of H_2_O_2_, robust production of O^•−^_2_ was also observed after addition of Fe^2+^ to HRP (Figure 3). Note that the addition of Fe^2+^ resulted in biphasic increase in CLA-CL, consisting of an immediate short-lasting spike followed by secondary but intense and long-lasting peak depending on the concentration of Fe^2+^ added. As far as we understand, the initial spikes sized similar to the water control are kinds of artifacts by rapidly injecting the reagent or control water through a syringe which rapidly causes the mixing of media and air (containing oxygen). This type of spikes can be commonly observed for CLA-CL monitoring as described in our previous studies (Monetti et al., 2014). In fact, the increase higher than the level of water control could have been attributed to the action of Fe. Therefore, there would be two modes of oxidative burst induced by ferrous ion, one is rapidly induced by lower range of Fe^2+^ concentrations observed as the short-lasting increase in initial spike of CLA-CL, and another follows the initial spike, gradually attaining much higher peak level of CLA-CL by responding to relatively higher range of Fe^2+^ concentrations (Figure 3A).

Here, we emphasized the secondary peaks which last for more than some minutes. Interestingly, depending on the concentrations of Fe^2+^ added to HRP, both the extent and duration of oxidative burst largely varied. Furthermore, we observed that successive additions of Fe^2+^ (6 times an hour) repeatedly caused the burst of O^•−^_2_ by HRP until the CL probe was completely consumed (Figure 3C), suggesting that the enzyme has capacity for continuous and robust oxidative burst if reducing agents are continuously supplied.

**Figure 4:**
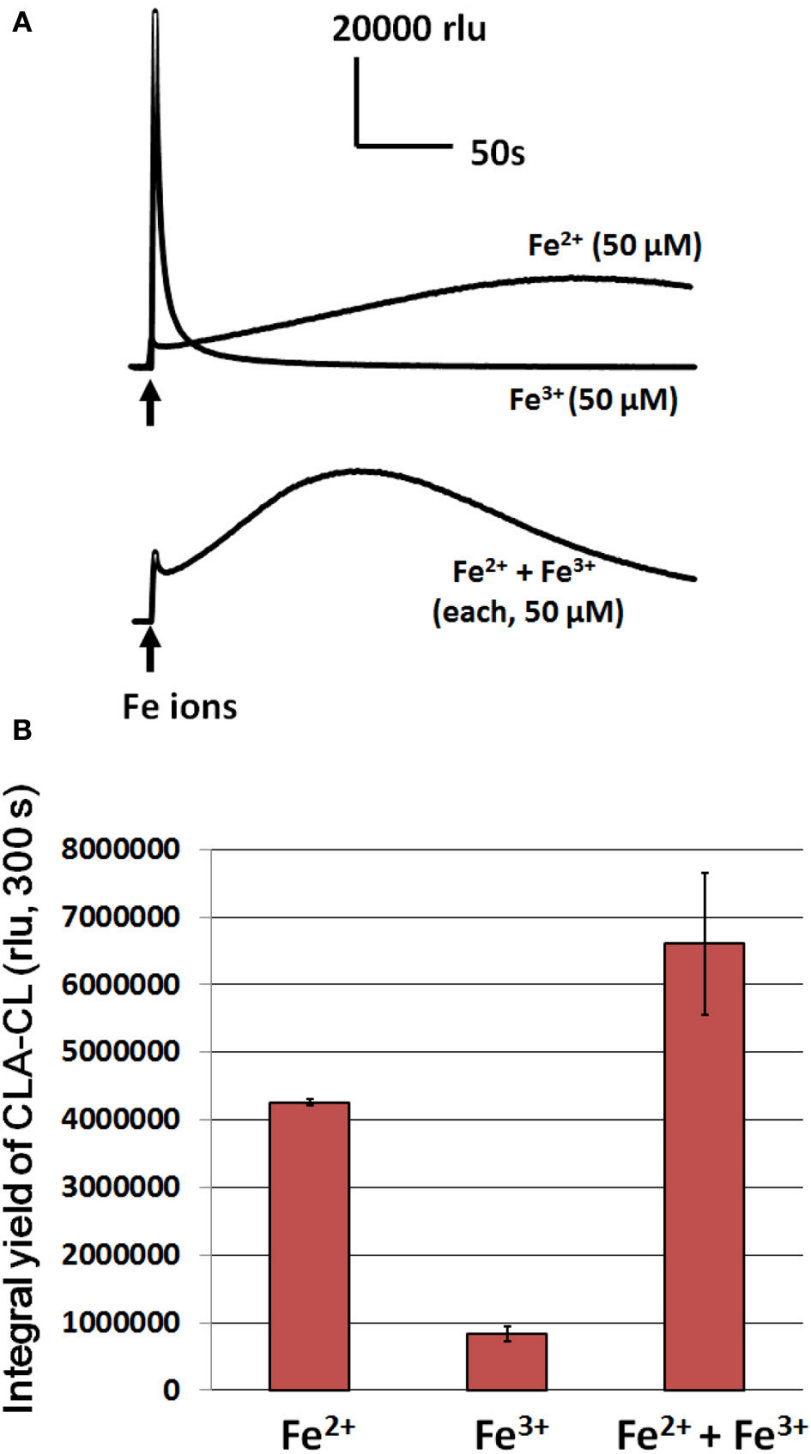
**Effects of ferrous and ferric ions on induction of O^•^_2_ generation in HRP reaction mixture. (A)** Temporal changes in O^•^_2_-dependent CLA-CL following addition of 50 μM Fe ions. **(B)** Effect of differently supplied Fe ions on the integral yield of CLA-CL (within 300 s) are compared. Conditions: total volume, 0.2 ml; K-phosphate, 25 mM (pH 7.0); HRP, 1.5 μM; CLA, 4 μM.

As shown in Figure 4, both ferric and ferrous ions induce the generation of O^•−^_2_. However, the temporal profiles of ferric and ferrous-induced O^•−^_2_ generation largely differed, suggesting that the modes of O^•−^_2_ generation may also differ (at present, such difference is unknown). The Fe^2+^-induced O^•−^_2_ generation last for 5–10 min while the Fe^3+^ induces a short-lived spike of O^•−^_2_ generation only. By comparing the yield of CLA-CL (within 300 s), the extent of Fe^2+^-induced O^•−^_2_ generation is at 5-fold greater level compared to the Fe^3+^-induced one (Figure 4B). Addition of Fe^2+^/Fe^3+^ mixture induced a compromised pattern of CLA-CL (Figure 4A), with much higher yield of CLA-CL (Figure 4B). These data are indicative of the potential impact of free iron ions both at ferric and ferrous state may stimulate the oxidative burst mediated by plant POX although we focus mostly on the action of ferrous ion in the present study.

### Involvement of O_2_ in Fe^2+^-induced HRP reaction leading to O^•−^_2_ generation

**Figure 5:**
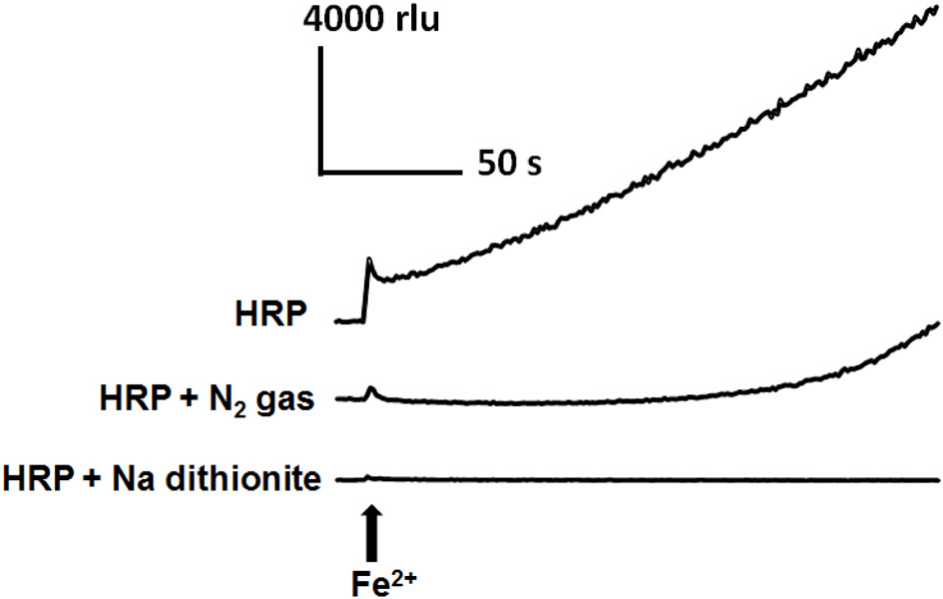
**Involvement of dissolved O_2_ on Fe^2+^-induced O^•^_2_ generation in HRP reaction mixture**. Increase in CLA-CL induced by 50 μM Fe^2+^ was inhibited by two distinct de-oxygenating treatments, namely, by bubbling with N_2_ gas (30 s) and addition of 20 mM sodium dithionite (sodium hydrosulfite). Conditions: total volume, 0.2 ml; K-phosphate, 25 mM (pH 7.0); HRP, 1.5 μM; CLA, 4 μM.

As the involvement of dissolved oxygen was suggested by preliminary spectroscopic monitoring of N_2_-sensitive conversion of Fe^2+^-treated native enzyme into CII, we examined the requirement for O_2_ in Fe^2+^-induced HRP reaction leading to O^•−^_2_ generation (Figure 5). The Fe^2+^-induced increase in CLA-CL was effectively lowered or delayed by removal of dissolved oxygen by N_2_ bubbling and inhibited by addition of sodium dithionite which is a convenient reagent that rapidly removes dissolved oxygen. As the air diffuses back into the reaction mixture even after replacement with N_2_ gas by bubbling, the state of inhibition due to the lack of O_2_ did not last long. On the other hand, chemically performed deoxygenation by Na dithionite resulted in clear-cut inhibition.

Above data are in support of our working hypothesis that, similarly to IAA-responsive mechanism, Fe^2+^-mediated conversion of native POX into ferrous enzyme intermediate further reacts with O_2_ to form catalytically inactive CIII which readily dissociates and releases O^•−^_2_ and native enzyme (Figure 1D).

### Effect of pH on HRP-catalyzed oxidative burst

**Figure 6:**
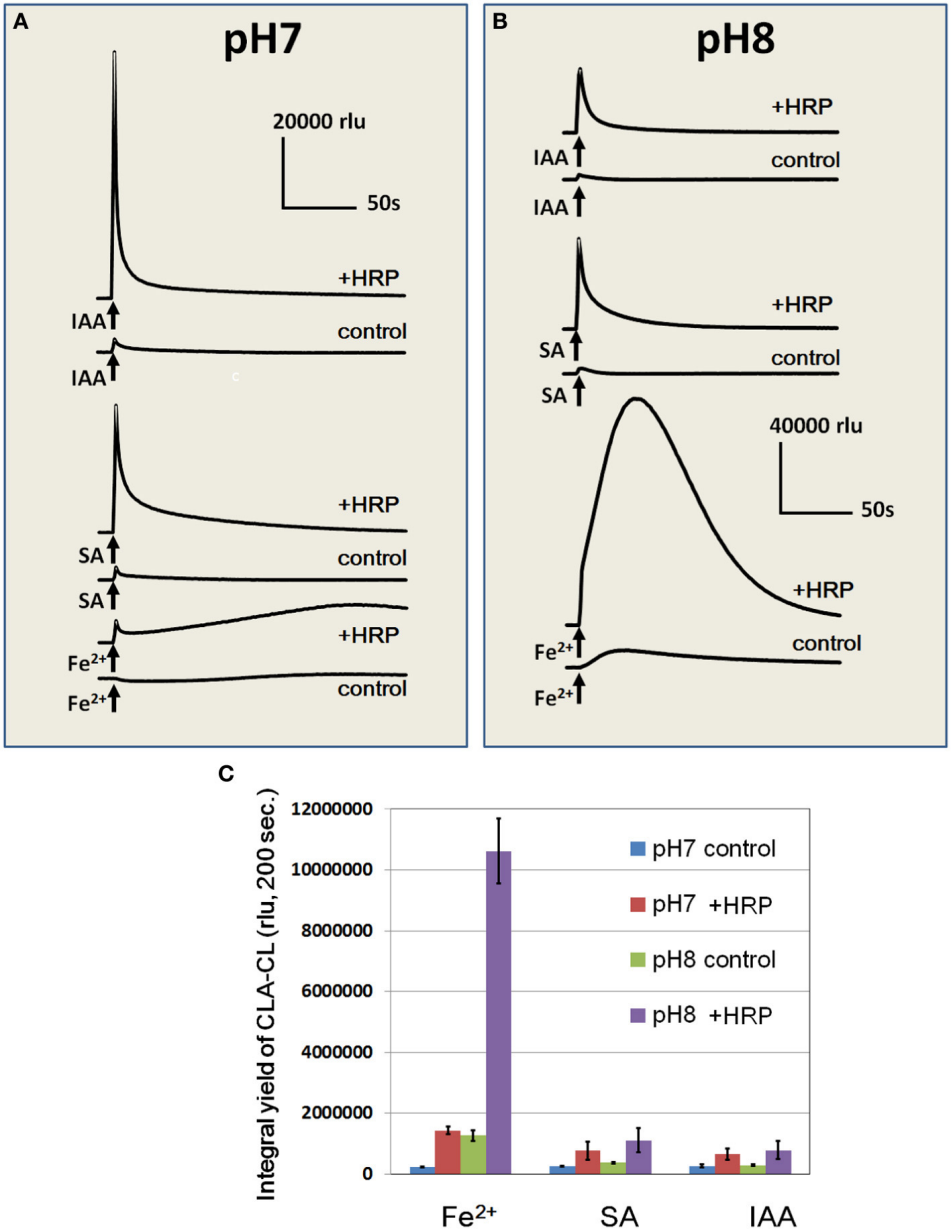
**Effects of Fe^2+^, SA, and IAA on O^•^_2_ generation in HRP reaction mixture at pH 7.0 and 8.0**. Fe^2+^, SA, or IAA (each 50 μM) was added to control buffer or HRP reaction mixture at pH 7.0 **(A)** and 8.0 **(B)**. **(C)** Integral yields of CLA-CL induced by Fe^2+^, SA, and IAA under different pH were compared. Error bats, SD (*n* = 3). Conditions: total volume, 0.2 ml; K-phosphate, 25 mM (pH 7.0 or 8.0); HRP, 1.5 μM; CLA, 4 μM.

Effects of Fe^2+^ (50 μM) on induction of O^•−^_2_ generation was compared with two known inducers of POX-mediated oxidative burst, namely, SA and IAA (each 50 μM) under neutral and alkaline pH (pH 7.0 and 8.0; Figure 6). Among three O^•−^_2_ inducers, IAA was shown to be most active in induction of the spike of O^•−^_2_ while Fe^2+^ was the only chemical active in stimulation of a gradual and long-lasting mode of O^•−^_2_ production. As reported (Kawano and Muto, 2000; Takayama et al., 2012), SA-induced production of O^•−^_2_ was not impressive in the absence of initial H_2_O_2_ supplementation.

**Figure 7:**
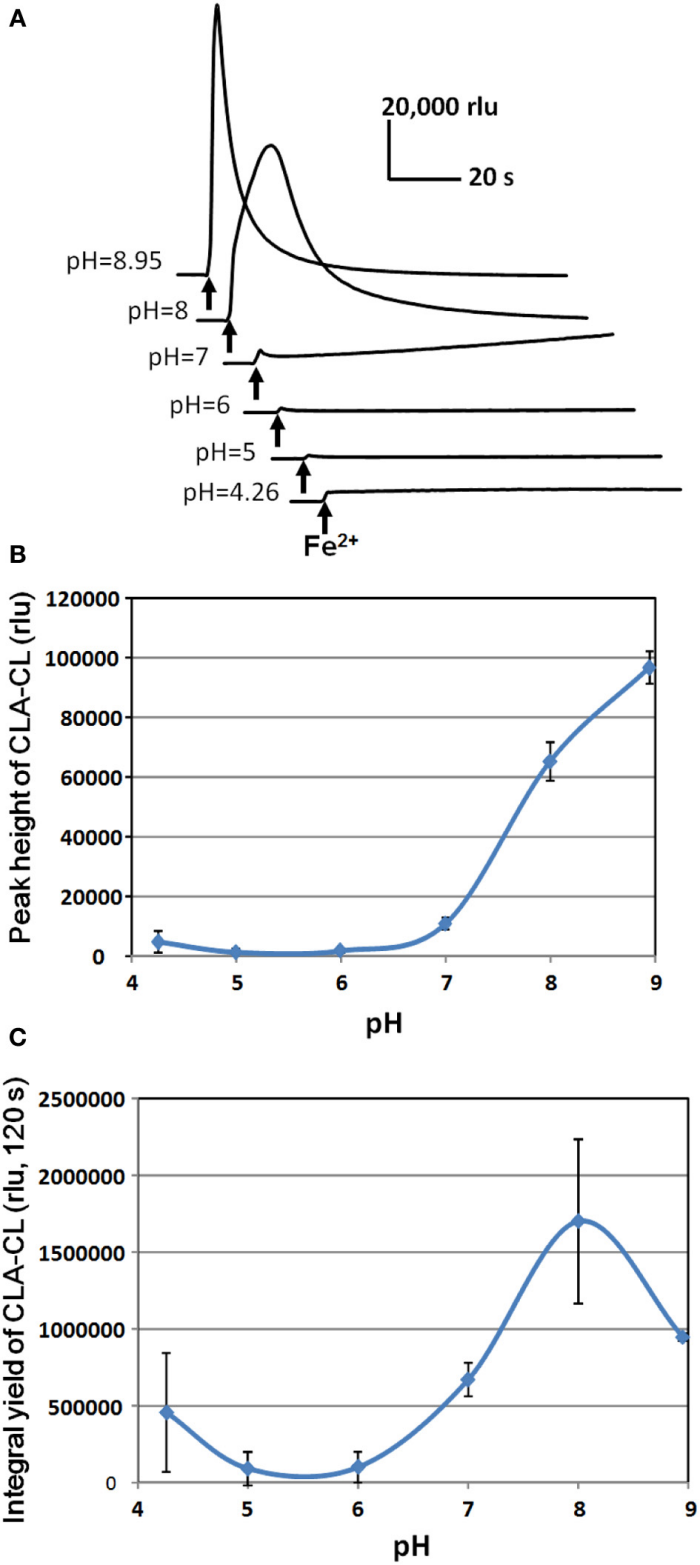
**Effect of pH on Fe^2+^-induced O^•^_2_ generation in HRP reaction mixture. (A)** Typical traces of Fe^2+^-induced CLA-CL recorded at various pH. **(B)** pH-dependent change in the peak height of CLA-CL. **(C)** pH-dependent change in the integral yield of CLA-CL (within 120 s after addition of Fe^2+^). Error bars, SD (*n* = 3). Conditions: total volume, 0.2 ml; K-phosphate, 25 mM (at indicated pH); Fe^2+^, 50 μM; HRP, 1.5 μM; CLA, 4 μM.

Despite of difference in the temporal profiles of induced O^•−^_2_ generation, the cumulative yields of O^•−^_2_ in response to Fe^2+^, SA, and IAA under neutral condition (pH 7.0) were at similar level (Figure 6C). It is noteworthy that the burst of Fe^2+^-stimulated O^•−^_2_ generation drastically increased under alkaline condition (pH 8.0) while SA and IAA showed no significant pH response (Figures 6B,C). Effect of pH on the Fe^2+^-induced O^•−^_2_ generation in HRP reaction mixture was further assessed by altering the medium pH between pH 4.26 and 8.95 (Figure 7). In the alkaline range (pH >7.0), the height of CLA-CL was shown to be drastically elevated. However, integral yield of CLA-CL was slightly lowered at highest pH examined as the pattern of O^•−^_2_ generation likely becomes spiky and less sustainable as pH elevated.

Above data suggested that HRP H_2_O_2_-independently catalyzes the production of O^•−^_2_ from dissolve oxygen in the presence of ferrous ion. This model is distinct from the previously known mode of ROS production catalyzed by plant POXs. Therefore, in the below section, we wish to review and compare the likely mechanisms.

## Discussion

Interestingly, nitric oxygen (NO) is one of known agents that bind and convert the ferric form of hemoproteins such as non-symbiotic hemoglobin from *Arabidopsis* (Perazzolli et al., 2004) into ferrous hemoproteins. In cases of plant POXs such as of soybean, the consequence of exposure of native POX to gaseous NO was accumulation of CII without exogenous supplementation of H_2_O_2_, suggesting that protein is once converted to ferrous form and eventually converted to and arrested as CII (Takayama et al., 2012). Among the known intermediates of POX and hemoproteins, the intermediate with ferrous heme is the only form with affinity to molecular oxygen; therefore, we should consider the series of steps converting the native POX into ferrous enzyme, and involvement of dissolved oxygen, in order to obtain the CII.

By analogy to NO-responsive events, we attempted to propose a working hypothesis explaining the paths of reactions Fe^2+^-dependently converting the native POX into CII in the absence of exogenous supplementation of H_2_O_2_ (Figure 1D). Firstly, the Fe^2+^-mediated conversion of native POX into ferrous enzyme intermediate occurs. Then, this intermediate molecule further reacts with O_2_ to form catalytically inactive CIII which readily dissociates and releases O^•−^_2_ and native enzyme. Eventually, CII can be formed through interaction between the native enzyme and H_2_O_2_ which is derived from O^•−^_2_. Note that the O^•−^_2_-to-H_2_O_2_ conversion can proceed in the presence of hemoproteins and non-heme free iron ions as discussed later.

Our working hypothetic model can be divided into two phases. In the first phase, conversion of native enzyme into ferrous intermediate must be caused so that finally resulting in production of O^•−^_2_ upon interaction with molecular oxygen (oxygenation cycle). After completing this cycle, the enzyme must go into further cycles if excess of Fe^2+^ is present. In the second phase, supply of H_2_O_2_ possibly derived from O^•−^_2_ must occur in order to fuel the conventional POX cycle.

Therefore, we have carried out (a) spectroscopic analysis of the fate of native HRP following the addition of Fe^2+^, (b) direct measurements of Fe^2+^-induced oxidative burst represented by generation of H_2_O_2_ and O^•−^_2_ and (c) examinations on the involvement of molecular oxygen in (a) and (b).

### POX-catalyzed ROS production involving hydrogen peroxide

The formulae shown below originally proposed for describing the mechanism for SA-dependent generation of O^•−^_2_ in plant system (Kawano et al., 1998; Kawano and Muto, 2000) suggest that the byproducts of POX-catalyzed oxidation of phenolics are necessarily involved in generation of O^•−^_2_.

(1) $$\text{POX }{\text{N}}^{\left(3\right)}+{\text{H}}_{2}{\text{O}}_{2}\to \text{CI}{\;}^{\left(5\right)}+{\text{H}}_{2}\text{O}$$

(2) $$\text{CI}{\;}^{\left(5\right)}+\text{AH}\to \text{CII}{\;}^{\left(4\right)}+{\text{A}}^{•}$$

(3) $$\text{CII}{\;}^{\left(4\right)}+\text{AH}\to \text{POX N}{\;}^{\left(3\right)}+{\text{A}}^{•}$$

(4) $$2{\text{A}}^{•}+2{\text{O}}_{2}\to 2{\text{A}}^{+}+2{\text{O}}_{2}^{•-}$$

POX N stands for native ferric enzyme. A^•^ and A^+^ are free radical species and the two-electron oxidized intermediate product derived from substrate AH (such as phenolics or AMAs), respectively. The formal oxidation states of the heme within the enzyme are indicated by numbers in the small brackets. As above, phenolics form a group of e^−^ donating substrates while H_2_O_2_ is viewed as the only e^−^ acceptor. Then, phenoxy radical (shown as A^•^) released thereafter may react with molecular oxygen to form O^•−^_2_. Since O^•−^_2_ is readily transformed into H_2_O_2_ in biological systems, a single cycle of AH-oxidizing POX reactions initiated by single unit of H_2_O_2_ results in yield of two units of O^•−^_2_ which is equivalent to two units of H_2_O_2_, and therefore, by this way, ROS could be amplified (Kawano, 2003a).

In place of phenolics, AMAs could be used as another group of active substrates (Kawano et al., 2000a,b). Pinontoan and his colleagues have shown that aromatic monoamine-dependent oxidative burst can be widely observed not only in plants but also in yeast cells *in vivo* (Pinontoan et al., 2002) and pseudo-POX cycle of human hemoglobin (Kawano et al., 2002b).

In case of SA oxidation by plant enzymes, analytical data in support of the production of SA radical species (one of A^•^) has been obtained using electron spin resonance spectroscopy by employing a natural spin trapper, ascorbate (Kawano and Muto, 2000). After above studies, the likely structures of the radical and derived cationic intermediate were proposed by Gozzo (2003). In addition, the involvement of CI and CII as the intermediate species required for SA-dependent and AMA-dependent O^•−^_2_ generation was spectroscopically confirmed (Kawano et al., 2002c,d).

In plants, the SA-dependently produced O^•−^_2_ acts as a chemical signal required for development of defense mechanism against pathogenic microbes (Kawano et al., 1998) and closure of stomata on leaves (Mori et al., 2001; Khokon et al., 2011). In model plant cells, TPC1 calcium-permeable cation channel is a likely target of the SA signal transduction pathway mediated with O^•−^_2_ (Lin et al., 2005).

### H_2_O_2_-independent ROS production catalyzed by IAA-stimulated POXs

Metabolism of IAA is common interest to many plant biologists. Through oxidation of IAA *via* two different mechanisms, it has been considered that plant POXs are involved in the metabolism of IAA. One mechanism involves the conventional H_2_O_2_-dependent pathway and the other requires the incorporation of molecular oxygen (O_2_) but not of H_2_O_2_ (Gazaryan et al., 1996; Savitsky et al., 1999; Kawano et al., 2001). The conventional POX cycle for the oxidation of various substrates coupled to the consumption of H_2_O_2_ proceeds as follows:

(1) $$\text{POX N}{\;}^{\left(3\right)}+{\text{H}}_{2}{\text{O}}_{2}\to \text{CI}{\;}^{\left(5\right)}+{\text{H}}_{2}\text{O}$$

(5) $$\text{CI}{\;}^{\left(5\right)}+\text{S}\to \text{CII}{\;}^{\left(4\right)}+\text{P}$$

(6) $$\text{CII}{\;}^{\left(4\right)}+\text{S}+{\text{H}}^{+}\to \text{POX N}{\;}^{\left(3\right)}+{\text{H}}_{2}\text{O}+\text{P}$$

where S and P are the substrate and product of its one-electron oxidation, respectively (Kawano, 2003a).

IAA can be oxidized by a wide variety of plant POXs, as model has been proposed through the study using HRP focusing on the conventional H_2_O_2_-dependent reactions with no strict substrate specificity (Kawano, 2003a). It is noteworthy that most plant POXs including HRP oxidize IAA also *via* an alternative H_2_O_2_-independent pathway requiring O_2_ (Smith et al., 1982). Reportedly, unlike animal and microbial POXs, most members of plant POXs are considered to behave as highly specific IAA oxygenases by sharing the domains required for binding of auxin (Gazaryan et al., 1996). The proposed H_2_O_2_-independent cycle for IAA oxidation involves the formation of a ternary complex, enzyme-IAA-dioxygen (Savitsky et al., 1999), finally yielding IAA cation radicals and O^•−^_2_ as by-products as follows (Kawano et al., 2001):

(7) POX+IAA↔[POX-IAA]

(8) $$\left[\text{POX-IAA}]+{\text{O}}_{2}↔[{\text{POX-IAA-O}}_{2}\right]$$

(9) $$[{\text{POX-IAA-O}}_{2}]\to \text{POX}+{\text{IAA}}^{+}+{\text{O}}_{2}^{•-}$$

where IAA^•+^ stands for IAA cation radicals. As above, plant POXs can catalyze the IAA-dependent generation of O^•−^_2_ in the absence of H_2_O_2_. However, depending on the concentrations of ROS and IAA, plant enzymes are readily inactivated and degraded by forming P-670 pigment which is an irreversibly inactivated form (Kawano et al., 2002a).

Based on the views that formation of enzyme-substrate complexes such as [POX-IAA-O_2_] results in release of O^•−^_2_ (Kawano et al., 2001), medical application of HRP-labeled antibodies and IAA has been proposed as a novel O^•−^_2_-generating system for cancer cell-targeted and controlled cell death induction, by designing the HRP-conjugated immuno-labeling of cancer-related molecules or expression of recombinant HRP in mammalian cells (Folkes and Wardman, 2001; Folkes et al., 2002; Kawano, 2003b; Dai et al., 2012). Although the IAA-induced O^•−^_2_ in HRP reaction mixture is very intense, the IAA-induced oxidative burst likely lasts only for few seconds (Kawano et al., 2001). This is largely due to the fact that IAA behaves as a suicide substrate for plant POXs, in its excess, irreversibly converting the oxygen-dependently formed CIII into inactivated verdohemoprotein (P-670) (Kawano et al., 2002a). In this point of view, induction of robust and long-lasting oxidative burst by application of Fe^2+^ may expand the possible applications of plant POXs for medical purposes.

### Possible roles for ferrous POX intermediate and CIII

As reviewed elsewhere, the conventional POX cycle involves the formation of CI in which the localization of second radical could be on the heme or on amino acid residues around the heme pocket depending on the nature of protein species (Kawano, 2003a,b). This variation may largely contribute to determination of the types of reaction catalyzed by hemoproteins. On the other hand, we have previously proposed the hypothetical reactions in the oxygenation cycle of plant POXs (Figure 1A) which can be solely attributed to the chemistry of heme, by analogy to the behaviors of other hemoproteins such as hemoglobin (Kawano et al., 2004).

CIII is the temporally inactive POX intermediate (heme-Fe^II^-O_2_) which is analogous to oxygen-bound hemoglobin which is readily auto-oxidized and decomposed into O^•−^_2_ and met-hemoglobin (Arayash, 1999; Kawano et al., 2002b). Note that met-hemoglobin is a ferric protein analogous to the native ferric POX and interestingly, pseudo-peroxidase catalytic activity can be found in met-hemoglobin (Kawano et al., 2002b).

When the heme-oxygen complex in CIII of plant POX dissociates, O^•−^_2_ and the native enzyme are released. Many of heme proteins, such as POX from French bean, are capable of generating H_2_O_2_ (derived from O^•−^_2_) at higher pH by a mechanism that involves the formation of CIII (Bolwell et al., 2002), thus distinct from the conventional POX reaction-mediated oxidative burst as such involving SA (Kawano et al., 1998).

The most likely mechanism considered to form CIII is the direct conversion of ferric proteins into CIII *via* single step in the presence of O^•−^_2_: heme-Fe^III^ + O^•−^_2_ → heme-Fe^II^-O_2_ or heme-Fe^III^-O^•−^_2_ (Arayash, 1999). However, this model fails to explain the burst of O^•−^_2_ often observed in plant POX model (Bolwell et al., 1998, 2002).

The missing link in the oxygenation cycle of ROS production involving CIII is the mechanism for reduction of native ferric POX (heme-Fe^III^) to ferrous enzyme (heme-Fe^II^). It is tempting to hypothesize that pH-dependent CIII-mediated ROS production requires the formation of CIII *via* preceding formation of ferrous enzyme, thereafter allowing spontaneous oxygenation into CIII (heme-Fe^II^ + O_2_ → heme-Fe^II^-O_2_) as predicted earlier (Kawano et al., 2004). Therefore, apparently, the formation of ferrous enzyme from native POX would be an important step for supporting the oxidative burst involving CIII.

It is well documented that extracellular alkalization occurs in plant tissues upon attacked by pathogens or treatments with elicitors, eventually allowing the pH-dependent extracellular POX-mediated oxidative burst at the site of microbial challenges (Bolwell et al., 1998). Here, we would like to propose a likely role for free ferrous ion in reduction of ferric POX into ferrous enzyme which readily produces O^•−^_2_ *via* mechanism involving CIII, especially under alkaline condition possibly contributing to the plant defense mechanism.

In conclusion, the present study proposed that plant POX-catalyzed production of O^•−^_2_ can be stimulated in the absence of conventional POX substrates but in the presence free Fe^2+^ through following reactions (Figure 1D):

(10) $$\text{POX N}{\;}^{\left(3\right)}+{\text{Fe}}^{2+}↔\text{ferrous-POX}{\;}^{\left(2\right)}+{\text{Fe}}^{3+}$$

(11) $$\text{ferrous-POX}{\;}^{\left(2\right)}+{\text{O}}_{2}↔\text{CIII}{\;}^{\left(6\right)}$$

(12) $$\text{CIII}{\;}^{\left(6\right)}↔\text{POX N}{\;}^{\left(3\right)}+{\text{O}}_{2}^{•-}$$

We view here that the recorded H_2_O_2_ could be derived from O^•−^_2_. Recent study provided us a notation that iron peroxide species have been identified as important intermediates in a number of nonheme iron as well as heme-containing enzymes (Namuswe et al., 2008). Therefore, by analogy, we could possibly expect that ferrous intermediate of plant POXs also interact with O^•−^_2_ to yield H_2_O_2_ in a manner similar to bacterial superoxide reductase. Or, excess of Fe^2+^/Fe^3+^ unbound to enzymes also non-enzymatically catalyses the disproportionation of O^•−^_2_ into H_2_O_2_.

Eventually, resultant H_2_O_2_ may contribute to conversion of native POX to CII *via* CI as previously demonstrated (Kawano et al., 2002c,d) (Figure 1D). Conversion of native POX to CII in the absence of initial supply of H_2_O_2_ reportedly occurs by treating soybean POX with NO (Takayama et al., 2012). Since NO converts some heme enzymes such as catalase into CIII (unpublished results), the mechanisms undermined should be similar to the case of Fe^2+^-induced redox changes in HRP.

### Conflict of interest statement

The authors declare that the research was conducted in the absence of any commercial or financial relationships that could be construed as a potential conflict of interest.
